# Nutritional Correlates of Koala Persistence in a Low-Density Population

**DOI:** 10.1371/journal.pone.0113930

**Published:** 2014-12-03

**Authors:** Eleanor Stalenberg, Ian R. Wallis, Ross B. Cunningham, Chris Allen, William J. Foley

**Affiliations:** 1 Division of Evolution, Ecology and Genetics, Research School of Biology, The Australian National University, Canberra, Australian Capital Territory, Australia; 2 Fenner School of Environment and Society, The Australian National University, Canberra, Australian Capital Territory, Australia; 3 National Parks and Wildlife Service, NSW Office of Environment and Heritage, Far South Coast, New South Wales, Australia; University of Tasmania, Australia

## Abstract

It is widely postulated that nutritional factors drive bottom-up, resource-based patterns in herbivore ecology and distribution. There is, however, much controversy over the roles of different plant constituents and how these influence individual herbivores and herbivore populations. The density of koala (*Phascolarctos cinereus*) populations varies widely and many attribute population trends to variation in the nutritional quality of the eucalypt leaves of their diet, but there is little evidence to support this hypothesis. We used a nested design that involved sampling of trees at two spatial scales to investigate how leaf chemistry influences free-living koalas from a low-density population in south east New South Wales, Australia. Using koala faecal pellets as a proxy for koala visitation to trees, we found an interaction between toxins and nutrients in leaves at a small spatial scale, whereby koalas preferred trees with leaves of higher concentrations of available nitrogen but lower concentrations of sideroxylonals (secondary metabolites found exclusively in eucalypts) compared to neighbouring trees of the same species. We argue that taxonomic and phenotypic diversity is likely to be important when foraging in habitats of low nutritional quality in providing diet choice to tradeoff nutrients and toxins and minimise movement costs. Our findings suggest that immediate nutritional concerns are an important priority of folivores in low-quality habitats and imply that nutritional limitations play an important role in constraining folivore populations. We show that, with a careful experimental design, it is possible to make inferences about populations of herbivores that exist at extremely low densities and thus achieve a better understanding about how plant composition influences herbivore ecology and persistence.

## Introduction

It is widely postulated that nutritional factors drive bottom-up, resource-based patterns in herbivore ecology, distribution and abundance [1], [2]. The nutritional quality of leaves for herbivores is largely determined by leaf chemistry and is influenced by the complex interactions between the types and amounts of nutrients and toxic chemicals in leaves. Although it is clear that leaf chemistry impacts the feeding behaviour of captive herbivores [3]–[6], it is still unclear how free-living herbivores respond to variations in leaf chemistry. Leaf chemistry can vary at different spatial scales, from tree-to-tree variations between and among species [7] to larger-scale patches of high and low nutritional quality habitats across a landscape [8], [9]. Foraging behaviours of animals are also scale-dependent, where folivores make small-scale decisions to choose desired individual trees and navigate at a larger scale between habitat patches [10], [11]. It is often assumed that factors that determine small-scale habitat choices will influence the larger scale movements and ecology of animals; however few studies have examined this question [12], [13].

Animals simultaneously need many different nutrients. Optimal foraging theory predicts that animals choose foods in order to maximise their intake of energy-rich substrates per unit time feeding [14], [15]. However recent studies using the Geometric Framework [6], [16], [17] suggests that animals aim for an intake target that meets their requirement for protein, while satisfying the requirements for energy and other vitamins and minerals in the process. Compared to the diets of carnivores, plant parts contain low concentrations of essential nutrients such as amino acids (measured as nitrogen, N) and are simultaneously defended by a variety of potentially toxic plant secondary metabolites (PSMs). Toxic PSMs invoke metabolic costs either directly [5], [18], [19] or indirectly such as by forming indigestible complexes with nutrients to reduce their availability [5], [20]. Tannins in particular can reduce the availability of protein to animals [21], [22]. The use of tannin-blocking agents (such as polyethylene glycol 4000, PEG) with *in vitro* digestion of leaves has recently been suggested as a simple way to measure the proportion of the total foliar protein that an animal can digest, termed ‘available nitrogen’ or ‘available N’ [22]–[24]. Recent studies suggest available N could be an important factor limiting population densities of wild herbivores [25].

The ability of wild herbivores to tolerate, avoid or detoxify leaf chemicals while meeting their nutrient requirements is thought to ultimately determine their fitness [26]. Experimental studies of herbivores in captivity have revealed a wealth of information on the feeding behaviour of individual animals in response to specific leaf compounds. For example, no-choice feeding experiments of captive marsupial folivores have highlighted one group of toxic PSMs in eucalypts as particularly important feeding deterrents: the formylated phloroglucinol compounds (FPCs) [19], [27]–[32]. FPCs are present only in *Eucalyptus* species of the *Symphyomyrtus* subgenus [33], which is considered the preferred subgenus of our study organism, the koala (*Phascolarctos cinereus*) [34]. Captive animals will balance the toxic effects of PSMs like FPCs, terpenes and tannins against the benefits of obtaining nutrients by choosing nutrient-rich foods [32], reducing intake [19], [35], [36], increasing time between feeding bouts [30], [37] and by mixing food sources containing toxins metabolised by different pathways [20], [29], [31]. It is largely unknown, however, to what extent free-living folivores behave similarly and the consequences for folivore populations [38].

The koala is an iconic eucalypt specialist and has a varying population status across its range in eastern Australia and so is an excellent study organism to investigate the bottom-up effects of nutritional factors. The koala persists largely in declining or stable, small and low-density populations in the north of its range in New South Wales and south east Queensland [39]–[42]; while in the south, such as in Victoria and on offshore islands, there are many translocated populations of koalas that now persist at such high densities that they are considered pests [43], [44]. Many attribute these regional population trends to variation in the nutritional quality of the eucalypt leaves of their diet [45]–[47], but there is little evidence supporting this hypothesis. The difficulties and costs associated with large-scale field studies and subsequent chemical analyses are major barriers to research on folivores [48] and cause most researchers to disregard complex leaf chemistries and intraspecific differences and instead use tree species composition to define nutritional quality [46], [49]–[51]. Research on free-living koalas and other folivores is largely undertaken on high-density herbivore populations in higher-nutrient areas [52], [53]. In contrast, conservation and management efforts are focused on low-density, small and declining herbivore populations, often in low-nutrient areas [41], [44] and it is not known to what extent inferences drawn from previous research can be applied in these circumstances.

In this paper, we examine the influence of leaf chemistry on the distribution and ecology of a low-density population of koalas in south east New South Wales, Australia. We use koala faecal pellets as a proxy for visitation to trees. Using a nested experimental design, we sample trees at two spatial scales to investigate whether differences in leaf chemistry of neighbouring trees influence which trees koalas visit (within-plot), and whether differences in the leaf chemistry of trees in different areas influence which areas koalas visit (between-plot).

We hypothesise that:

(a) Koalas visit the trees with higher concentrations of foliar available N, leaf digestibility and (b) lower concentrations of FPCs when compared with leaves from a neighbouring tree;
(a) Koalas visit the trees with higher concentrations of foliar available N, leaf digestibility and (b) lower concentrations of FPCs when compared with leaves from a tree at a different plot.

Our findings will allow us to examine how the feeding behaviours shown by captive herbivores in short-term feeding experiments translate to the behaviour and ecology of free-living herbivores in low-nutrient habitats and provide insight into how natural variations in food quality limit wild herbivore populations.

## Methods

### Study site

We studied koalas in the forests between Bermagui and Tathra on the far south coast of New South Wales (36°26′ S, 150°00 E and 36°34′ S, 149°55′ E, 0–450 metres above sea level). The site (‘Bermagui-Mumbulla’) is approximately 22 000 hectares and includes National Parks, State Forests and private land. These dry, open sclerophyll forests are primarily on Ordovician metasediments with small areas of tertiary deposits and alluvial deposits in the river and valleys. Although once common, koalas are now considered to be locally rare [54], [55] and in the last 10 years have been recorded only in the northern hillside forests of the region [42].

The research was approved by the NSW Department of Primary Industries (Special Purpose Permit for Research R20/98) and authorized under the NSW National Parks and Wildlife Act 1974, s132c, (Scientific License 101079). Access and sample collection on private land was approved by the land owners.

### Spot Assessment Technique survey (SAT)

The site was initially surveyed for koala distribution between 2007 and 2009 using the Spot Assessment Technique (SAT) [56]. At every SAT plot, 30 neighbouring trees of any species over 150 mm diameter at 1.3 m (Diameter at Breast Height, DBH) were marked and then searched for two minutes for koala faecal pellets out to 1 m from the base of the tree. At completion of the SAT survey, 590 plots with 17 700 trees had been surveyed for pellets in a random grid pattern across the site. Only 60 SAT plots contained koala faecal pellets, suggesting that about 10% of the site was occupied by koalas at the time of this initial SAT survey. Although clustered in places, these occupied plots were widely scattered.

### Leaf collection protocol

**Figure 1:**
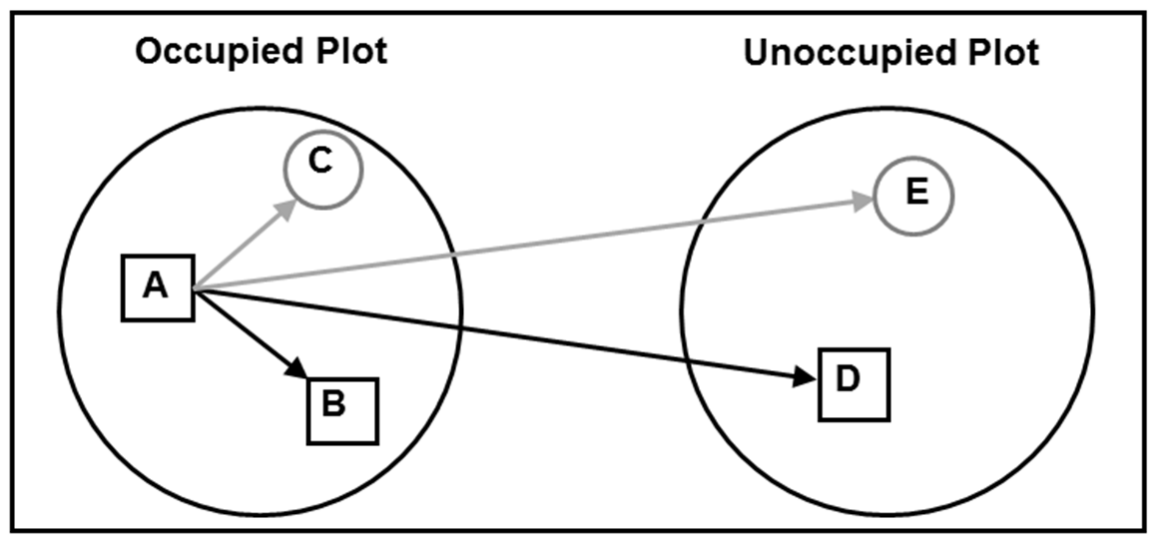
Schematic of nested sampling design with tree and plot categories. Experimental sampling design showing the tree and plot categories. At Occupied Plots, trees visited by koalas (A) were grouped with one nearby and similar tree that had not been visited (B) and with one nearby tree of a different subgenus (C). Then, at a matched Unoccupied Plot, leaves were collected from a tree of the same species (D) and one of a different subgenus (E) to Category A trees.

Trees were sampled for this study from October to November 2009. Using a nested experimental design (Figure 1), we randomly selected 20 SAT plots where koala faecal pellets had been found (‘occupied’ plots) and paired each of these with a plot within 1 km with similar tree species and elevation where no faecal pellets had been found (‘unoccupied’ plots). In the field at each occupied plot, we identified all 30 trees that were part of the initial SAT survey and identified those trees where koala faecal pellets had initially been found (visited trees) and trees where no pellets had been found (non-visited trees). From each visited tree, we collected 50 g of fully expanded, mature leaves without signs of insect infestation. We then collected leaves from two non-visited, neighbouring trees (i.e. no record of faecal pellets from the SAT plot) for each visited tree; the first was a tree of the same species, and the second from a different *Eucalyptus* subgenus (either *Symphyomyrtus* or *Eucalyptus* subgenus). To compare visited trees with non-visited non-neighbouring trees, we collected leaves from trees at the paired unoccupied plot, again collecting from a tree of the same species and from one of the different *Eucalyptus* subgenus for each visited tree. Thereby, every visited tree was grouped with four non-visited trees: two from the same occupied plot and two from the paired unoccupied plot (Figure 1). We searched all sampled trees for koalas, koala faecal pellets and measured DBH. After assessing that no koalas were nearby and that no other fauna were likely to be disturbed, leaves were collected from tree branches using a 12 gauge shotgun (MIROKU Model 10), a 0.204 calibre Ruger rifle (KIMBER Provarmint), or secateurs mounted on a 6 m telescopic aluminium pole. We placed leaves in individual paper bags and stored them in portable freezers.

### Leaf chemical analysis

We determined the concentrations of total nitrogen (total N), available nitrogen (available N), (the ranked effect of both tannins and fibre on leaf nitrogen concentration [23]), *in vitro* dry matter digestibility (DMD, a proxy for many nutrients) and FPCs in leaf samples from all sampled trees (n = 310).

Frozen leaf samples were freeze-dried and ground to pass a 1 mm sieve in a Cyclotec 1093 mill (Tecator, Sweden). We determined the concentrations of FPCs in all *Symphyomyrtus* samples (n = 168) following the method of Wallis and Foley [57]. The FPCs were extracted by sonicating 20±2 mg of freeze-dried, ground foliage with a known mass (ca 4.5 g) of solvent (7% water in acetonitrile containing 0.1% trifluoroacetic acid and 0.30 g per litre of the internal standard 2-ethylphenol) for five min. The mixture was filtered (0.22 µm) into an autosampler vial and then 15 µL was injected onto a Wakosil 250×4 nm GL 3C18RS (SGE Analytical: Ringwood, Australia) column maintained at 37°C with a flow rate of 0.75 mL/min on a Waters Alliance Model HPLC. The FPCs were eluted under gradient conditions with 0.1% TFA acid in acetonitrile (A) and 0.1% TFA in water (B) as follows: 60% A/40% B for 5 min, linear gradient to 90% A 10% B at 60 min, held for 10 min and returned to starting conditions over 10 min. We measured the peak response at 275 nm and calibrated the concentration with standards purified in the laboratory.

We selected a representative subset (n = 167) from all the leaf samples using their near-infrared reflectance spectra (NIRS) to analyse using an *in vitro* procedure [58]. The *in vitro* procedure involved incubation with polyethylene glycol (PEG), a tannin-blocking agent, together with pepsin and cellulase to rank trees with respect to the availability of N. The difference in the available N of samples digested with and without PEG is considered to be the effect of tannins. Using this method, we determined total leaf N, available N (the ranked effect of tannins and indigestible components of the diet on N), and *in vitro* DMD. We quantified the concentration of N in the original sample and the digested residues using the Dumas technique with a LECO TruSpec combustion N analyser (LECO Corporation, Michigan, USA) calibrated with EDTA. We calculated residual moisture by oven drying (60°C) 20 samples to a constant mass to express all results on a dry matter (DM) basis.

### Near Infrared Reflectance Spectroscopy

We obtained NIR spectra between 408–1093 nm, and between 1108–2493 nm of all 310 samples in duplicate using a scanning spectrophotometer with a spinning cup module (NIR System Model 6500, Foss, Silver Springs, Maryland, USA). We developed NIRS calibration equations from the *in vitro* subset (n = 167) to predict the foliar chemistry of the remaining samples. We randomly selected 20 samples to independently validate our NIRS predictions. All calculations used NIRS 3, version 4.00 (WinISI Infrasoft International, Port Matilda, Pennsylvania, USA). For most calibrations, we applied mathematical transformations of standard normal variate and detrend to raw near-infrared spectra to reduce the influence of particle size. We then used modified partial least squares regression and partial least squares regression with various combinations of Savitzy-Golay spectral-smoothing functions until the most robust equations were developed for each variable [59]. Relationships between NIR predicted values and the validation set were investigated using simple linear regressions and Pearson's correlation.

### Analyses and modelling of tree visitation

We fitted linear mixed models to the foliar compounds we measured using the residual maximum likelihood algorithm in GenStat 12^th^ Edition (VSN International, Ltd. Oxford, UK). This algorithm incorporates the fixed and random terms in the highly-nested study design to produce unbiased estimates of variance components and thus reduce the chance of type 1 error [60], [61]. We checked residuals for normality at each stage of the analysis.

We analysed each foliar compound individually by fitting Model (1) (below). This model incorporates all levels of nesting and spatial scale in the study design and therefore simultaneously investigates the relationships between leaf chemistry and koala visitation at the two spatial-scales. We fitted the model separately to plants from each subgenus to compare the chemistry of visited trees to non-visited trees of the same species both within and between-plots. To identify which species contributed to the broader pattern, we used Model (1) with restriction on each species. Non-significant terms were sequentially dropped from the models to obtain simplified models with only significant fixed terms, determined using a Wald test of significance.

Terms used in the model of fixed effect included tree activity, tree category, plot category, subgenus, and plot activity. Tree (A–D) and plot category (occupied or unoccupied) terms are defined in Figure 1. The presence of faeces at the base of trees was a proxy for koala visitation and determined the tree and plot activity level. Tree activity number represented the number of times koala faecal pellets were found at the base of the tree (visited twice, once or not at all). Plot activity represented the relative koala activity at each plot. It was calculated as the percentage of trees searched at each site (out of 30 trees) that had faecal pellets. The random model included terms for the plot pair and tree group.

Model (1).

Response: *Foliar compound*

Fixed model: *Constant + Plot activity + Plot type/Tree category + Tree activity + Subgenus*

Random model: *Plot pair/Plot type + Plot pair/Tree group*

Foliar total N, available N and DMD concentrations approximated a normal distribution within all but one species: *E. globoidea.*

## Results

### Visitation to different tree species

Koalas visited 67 trees of 8 eucalypt species: *E. longifolia*, *E. bosistoana*, *E. cypellocarpa*, *E. tricarpa* from the *Symphyomyrtus* subgenus; and *E. globoidea*, *E. muelleriana*, *E. agglomerata* and *E. sieberi* from the *Eucalyptus* subgenus. They visited trees of the *Symphyomyrtus* subgenus more than they did the *Eucalyptus* subgenus (40 versus 27) even though the *Eucalyptus* subgenus comprised 58% of the eucalypt trees at the occupied plots. Koalas tended to visit the most common species at the occupied plots: *E. longifolia* (visited n = 24), followed by the third most common species: *E. globoidea* (visited n = 11). They rarely visited *E. agglomerata* (visited n = 2). We found fresh koala faecal pellets under six new trees and at 29 trees where pellets were found during the original SAT survey. We thus concluded that koalas revisited 43% of the trees they had visited previously. Three koalas were sighted during the SAT field work between 2007 and 2009 but none during leaf collection.

### Near-Infrared Reflectance Spectroscopy

The NIRS calibration equations developed for all compounds measured with the *in vitro* analysis had R^2^ values between 0.95 and 0.97 and 1-VR values between 0.90 and 0.95 (Table 1). The predicted values were significantly correlated with analysed concentrations of the validation set (n = 20, P<0.001, Pearson's correlation coefficients for available N: 0.930; DMD: 0.890; available N in the presence of PEG: 0.954; DMD in the presence of PEG: 0.973; and total N: 0.962).

**Table 1:** Description of modified partial least squares regression equations relating near infrared spectra of *Eucalyptus* leaves to analytical values†.

Constituent	N	Mean	SD	R^2^	SECV	1-VR	Scatter	Data processing
Total N	162	1.06	0.16	0.97	0.04	0.95	SNV Detrend	2441
DMD with PEG	160	64.60	9.8	0.95	3.1	0.90	SNV Detrend	2641
DMD	159	65.50	9.4	0.96	2.6	0.92	None	2441
Available N with PEG	160	0.84	0.15	0.95	0.05	0.90	SNV Detrend	2441
Available N	160	0.72	0.24	0.96	0.07	0.92	SNV Detrend	2641

† N number of samples used in the equation, R^2^ the coefficient of determination between the spectra and the analytical values, SECV the standard error of cross validation, 1-VR coefficient of determination of cross validation; Scatter: no scatter correction or “standard normal variate and detrend”; “Data processing” provides details of the derivation and smoothing functions applied. For example, “2,4,4,1” refers to using the second derivative, leaving a gap of four wavebands between calculated values, doing a first smoothing over four wavebands and then a second smoothing over one waveband.

### Effects of leaf chemistry on koala preference

**Figure 2:**
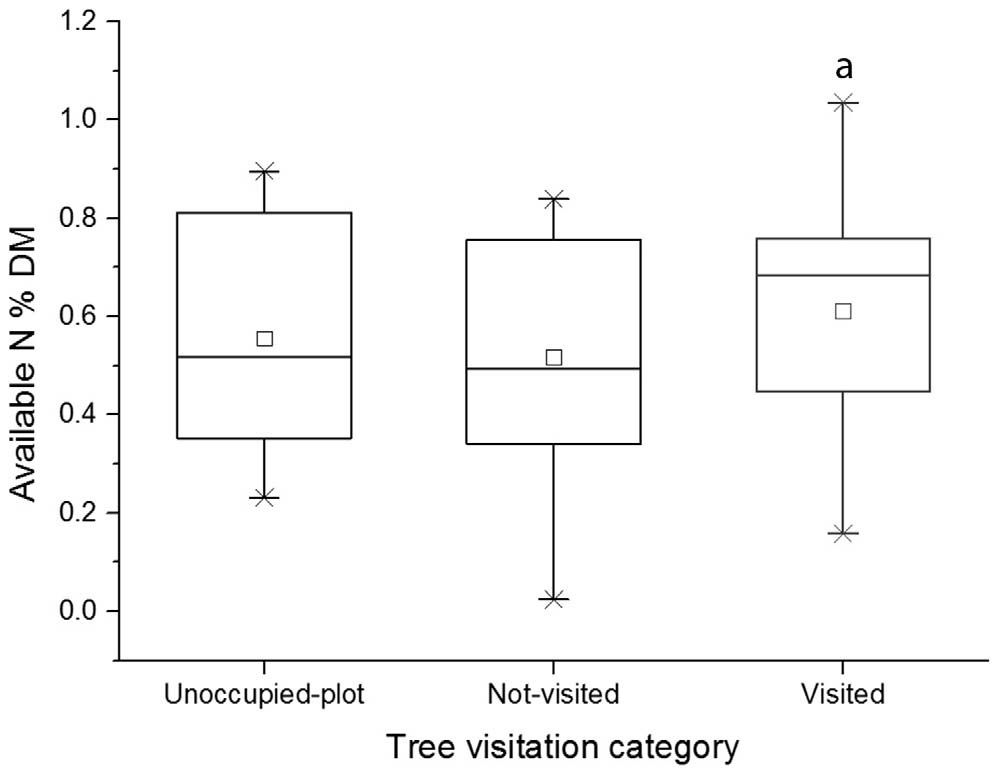
Available N concentrations in leaves from *Eucalyptus* subgenus trees in the REML model categories. Available N concentrations in the leaves of *Eucalyptus* subgenus trees in the three visitation categories used in the REML subgenus-specific model. Visited trees are trees that were visited by koalas, not-visited trees are neighbouring conspecifics from the same plot that were not visited by koalas, and unoccupied-plot trees are conspecifics from unoccupied plots. ^a^ Significantly different from other categories (P<0.05).

**Figure 3:**
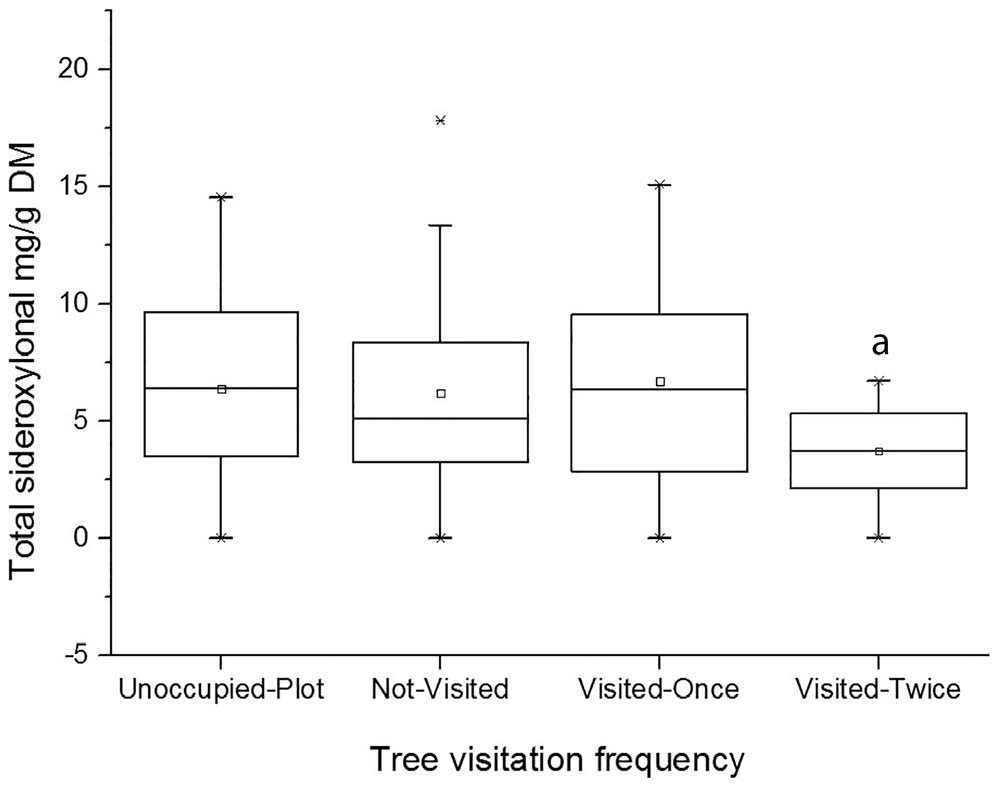
Sideroxylonal concentrations in leaves from *Symphyomyrtus* subgenus trees in the REML model categories. Sideroxylonal concentrations in the leaves of *Symphyomyrtus* subgenus trees in the four visitation categories used in the REML subgenus-specific model. The categories are trees that were visited twice by koalas (pellets found twice), trees that were visited once by koalas (pellets found once), neighbouring conspecifics from the same plot that were not visited, and conspecifics from unoccupied plots. ^a^ Significantly different from other categories (P<0.05).

The models revealed that koalas visited trees that had higher nutritional quality compared with a neighbouring tree of the same species (Tables 2 and 3). Koalas preferred trees of the *Eucalyptus* subgenus with higher concentrations of available N (16% higher, P = 0.003) and higher DMD (4% higher, P = 0.040) compared with concentrations in neighbouring conspecifics (Table 2, Figure 2), but were not influenced by the total N concentration in leaves. This effect was particularly evident among the *E. sieberi* trees visited by koalas, where the species-restricted model revealed a statistical significant difference between visited and non-visited trees (P = 0.005, N = 23, 9 visited). The *Symphyomyrtus* trees visited twice had foliage with significantly lower sideroxylonal concentrations compared with neighbouring conspecifics (41% lower, P = 0.016) (Table 3, Figure 3). Concentrations of the other FPCs, available N, total N and DMD were not significantly related to koala visitation to *Symphyomyrtus* trees, but the models suggested that the trees visited twice by koalas had lower total FPC and higher available N concentrations than did their neighbouring conspecifics (P = 0.077 and 0.094 respectively).

**Table 2:** Summary of REML model results for statistically significant foliar attributes in trees of the subgenus *Eucalyptus*.

*Eucalyptus* subgenus trees					
Foliar attribute	Predicted mean (± s.e.m. % DM) of trees visited at least once	% Difference of trees visited at least once to other categories	Wald statistic for fixed effects	d.f.	P-value†
Available N	0.61 (±0.03)	+16	9.93	1	0.003*
DMD	62.79 (±1.28)	+4	4.53	1	0.040*

† P-values that were statistically significant at P<0.05 are marked with asterisks.

**Table 3:** Summary of REML model results for statistically significant foliar attributes in trees of the subgenus *Symphyomyrtus*.

*Symphyomyrtus* trees					
Foliar attribute	Predicted mean (±s.e.m. mg/g DM) of trees visited twice	% Difference of trees visited twice to other categories	Wald statistic for fixed effects	d.f.	P-value†
Sideroxylonals	3.77 (±1.07)	−41	6.10	1	0.016*
Total FPCs	22.80 (±2.79)	−18	3.19	1	0.077
Available N	0.85 (±0.04)	+7	2.87	1	0.094

† P-values that were statistically significant at P<0.05 are marked with asterisks.

The leaf chemistry of trees in occupied plots did not differ significantly from the leaf chemistry of conspecifics in unoccupied plots. Variations in leaf chemistry were not related to differences in plot activity (how many trees were visited by koalas at each plot) or plot type (visited or non-visited) in the REML modelling. Therefore, leaf chemistry explained why koalas visited particular trees, although did not explain why koalas visited different locations.

### Comparisons of leaf chemistry between tree species

**Figure 4:**
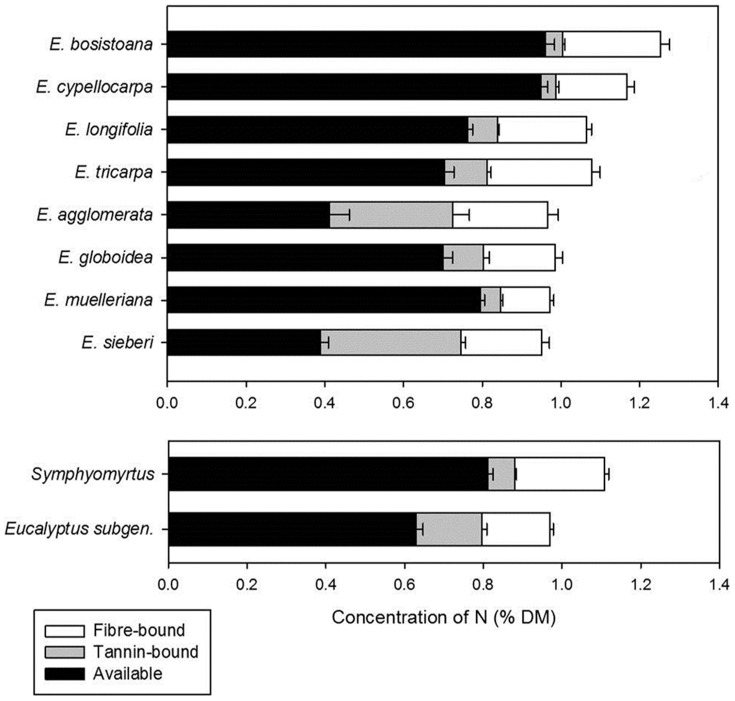
Components of total foliar N concentration for the two subgenera and each species. The proportions of the three chemical components that make up total foliar N concentration (% dry matter ± standard error of the mean, s.e.m.) in the two subgenera and in individual species. Total N is made up of the three components: proportion bound to indigestible fibre (shown in white), proportion bound to tannins (shown in grey) and the amount of N available to animals (available N, shown in black).

**Figure 5:**
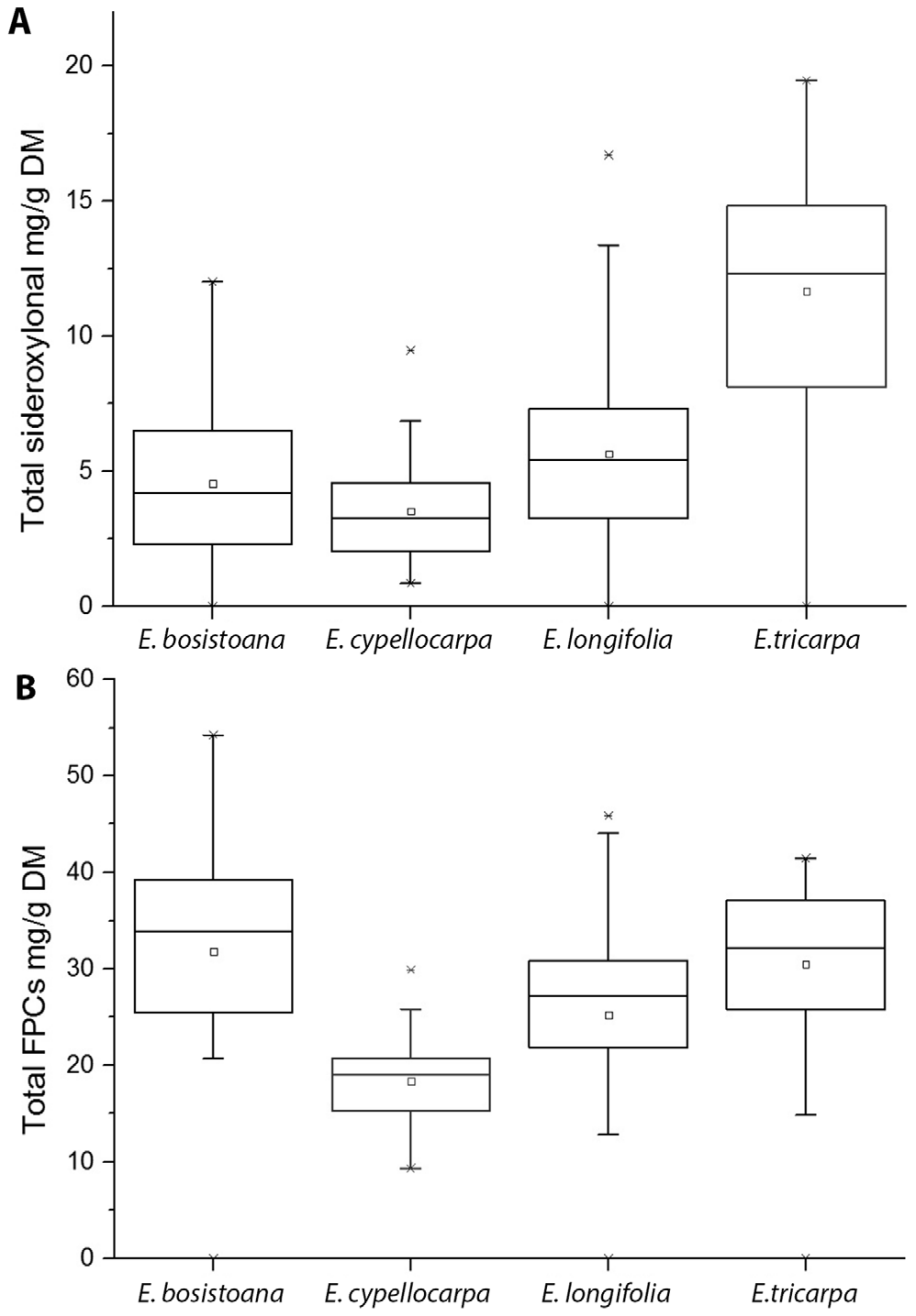
Mean concentrations of total sideroxylonal and total FPC concentrations in the four *Symphyomyrtus* species. Mean concentrations (± s.e.m.) of total sideroxylonal and Total FPCs in the four *Symphyomyrtus* species. Part A. Total sideroxylonal concentrations; Part B. Total FPC concentrations.

Species from the *Eucalyptus* subgenus had lower concentrations of available N in their leaves than did those from the *Symphyomyrtus*: however there was considerable between and within species variation. Total foliar N can be split into three components that show the ranked effect of tannins and fibre on N availability: available N, tannin-bound nitrogen and fibre-bound nitrogen (Figure 4). The leaves of two *Symphyomyrtus* species: *E. bosistoana* and *E. cypellocarpa* tended to have the highest available N, and the leaves of two *Eucalyptus* subgenus species: *E. agglomerata* and *E. sieberi* tended to have the lowest available N concentrations of the eight species. *Eucalyptus tricarpa* leaves tended to have higher concentrations of sideroxylonals and *Eucalyptus cypellocarpa* leaves had lower concentrations of sideroxylonals and total FPCs compared with the other *Symphyomyrtus* species (Figure 5).

## Discussion

Using a nested design that involved sampling at two spatial scales we show that koalas are influenced by an interaction between toxins and nutrients in leaves. Koalas visited trees with leaves containing higher available N and avoided trees with higher foliar sideroxylonal concentrations when compared with a neighbouring tree of the same species. This result supports hypotheses 1) (a) and (b) and suggests that koalas in low-nutrient habitats prioritise nutritional concerns when selecting individual eucalypt trees to visit. In contrast, the leaf chemistry of visited trees at occupied plots did not differ from that of paired trees at the unoccupied plot, suggesting that leaf chemistry did not influence the koalas at this broader spatial scale and refuting hypotheses 2) (a) and (b).

### Diet selection by free-living herbivores

The Bermagui-Mumbulla site encompasses typical eucalypt forests that contain a mixture of species from the two main eucalypt subgenera – *Symphyomyrtus* and *Eucalyptus*. *Symphyomyrtus* species have leaves that contain FPCs and high concentrations of these compounds are toxic to herbivores [62]. In contrast, species from the *Eucalyptus* subgenus do not produce FPCs but the leaves contain lower concentrations of available N and more indigestible material than do those of *Symphyomyrtus*. In both subgenera, leaf chemistry was highly variable among trees of the same species [33], [48] and folivores may take advantage of this intraspecific variation to meet their nutritional requirements from the available trees [5], [19]. When visiting species from the *Eucalyptus* subgenus, koalas selected individual trees with higher foliar concentrations of available N and DMD, although not total N, when compared with a neighbouring tree of the same species. In contrast, variations in concentrations of a single FPC, sideroxylonal, determined visits by koalas to *Symphyomyrtus* trees.

Our results reveal a tradeoff between toxins and nutrients in diet choice. The consumption of leaves from trees containing lower concentrations of available N is driven by the avoidance of FPCs; while in turn, the consumption of leaves from trees with higher concentrations of available N is driven by the need to avoid consuming large quantities of gut-filling indigestible tissues before nutrient targets are met [63]–[65]. A series of studies on a free-living high-density population of koalas at Phillip Island in Victoria reveal a similar tradeoff between nutrients and toxins in koala feeding choices. Koala visitation to two tree species of the *Symphyomyrtus* subgenus was found to be related to low concentrations of total FPCs and higher total N concentrations [62], [66]. When feeding bouts were directly measured, researchers found that the koalas spent more time feeding in particular trees with higher concentrations of foliar available N when the concentration of FPCs was low, but had consistent short feeding bouts across different available N concentrations at high FPC concentrations [67]. The tradeoff between nutrients and toxins reflect the high protein and energy costs associated with detoxification and the limited capacity and efficiency of detoxification pathways [20], [68]. For example, the cost of detoxifying a single PSM, benzoate (a common plant secondary metabolite) was found to be about 20% of the total digestible protein intake of common brushtail possums [69]. This is a dramatic tax on protein intake and suggests that higher concentrations of available N in *Symphyomyrtus* may assist koalas to defray these costs [67].

Herbivores are limited in their ability to meet nutritional goals by their gut capacity, food retention times and the capacity and efficiency of detoxification pathways [20]; as well as by their ability to move safely and efficiency through the landscape [70]. In most cases, few individual trees have leaves that meet all the nutritional needs of a folivore at once and so animals must either accept the costs of a sub-optimal diet or switch to another food source to meet nutrient targets and avoid or dilute the effect of toxins [20]. Koalas are larger and less mobile than many other arboreal folivores and are particularly vulnerable to predation when moving on the ground between trees [71]. In captive feeding experiments, foraging efficiency and overall intake was increased when generalist herbivores were given access to a range of plants with diverse leaf chemical profiles in a small area [31], [72], [73]. Koalas are dietary specialists, however we found substantial differences in the amounts and types of chemical compounds in leaves of neighbouring *Eucalyptus* trees, even between trees of the same species, and we predict that this diversity provides koalas with sufficient choice to allow them to select a suitable and varied diet while minimising movement costs. This taxonomic and phenotypic diversity of trees is likely to be particularly important in low-quality habitats because there may be great distances between richer resources and so folivores are compelled to make direct tradeoffs to address immediate concerns at a small-spatial scale [4].

### Habitat quality influences herbivore populations

Demonstrating the link between nutritional constraints and population processes in wild herbivores remains a challenging but essential task. A major difficulty is the lack of a common currency and methodology in which to measure plant nutritional quality [38]. Ecologists have sought to define nutritional quality using a variety of metrics such as soil fertility [9], [74], leaf protein concentrations [9], [46], and a ratio of leaf protein to fibre concentrations [75]. These simple metrics and ratios fail to adequately capture the complex plant chemistries in the food of mammalian herbivores, in particular the effect of tannins on the availability of protein [22]. In contrast, available N accounts for the multivariate nature of herbivore nutrition and so is a more appropriate method to describe food quality from an herbivore's perspective [22], [23].

Our study allowed us to make inferences about the role of leaf chemistry in influencing low-density herbivore populations and thus contributes to our understanding of how nutrition regulates populations of herbivorous mammals. Our foliar available N assays support the view that Bermagui-Mumbulla is of lower nutritional quality than other parts of the koala's range. The occupied plots were dominated by the species of the *Eucalyptus* subgenus that contain lower concentrations of available N. *Eucalyptus longifolia* was the dominant species of the *Symphyomyrtus* subgenus and showed only moderate available N values. Trees from the subgenus *Eucalyptus* visited by koalas had leaves with mean available N concentrations of 0.61±0.03% DM. In contrast, trees of the *Symphyomyrtus* subgenus that were visited had mean foliar available N concentrations of 0.85±0.04% DM. In comparison, Marsh and colleagues [67] found that trees visited by koalas at Phillip Island had available N concentrations above 1.15% DM. Small differences in foliar available N in trees available to wild folivores have been shown to have significant impacts on population dynamics and reproduction. For example, common brushtail possums (*Trichosurus vulpecula*) inhabiting home ranges with low average concentrations of available N had lower reproductive success and slower growing offspring than did possums occupying home ranges with higher nutritional quality [25]. Similarly, McArt and colleagues [76] reported lower fecundity and twinning rates in moose where the available N concentrations of summer food was lower. The biomass of folivorous primate communities in Africa and Asia can been largely explained by the ratio of leaf protein-to-fibre [75], [77]–[79] but recent evidence suggests that tannins also play an important regulating role for folivorous primates [22], [80].

### Mechanisms regulating population expansion

The degree to which habitats will limit herbivore populations is determined by the overall quality and diversity of the trees available [4]. Animals in low-quality forests may be unable to obtain the necessary nutrients for population expansion, whereas low-diversity forests limit an herbivore's ability to select foods with different chemical profiles to achieve a varied diet. Digestion and PSM detoxification of low-quality leaf diets consumes more time, energy and nutrients when compared to a leaf diet that is rich in nutrients and low in fibre and toxins [5] and this additional time, energy and nutrients would otherwise be allocated to other activities such as social interaction and reproduction [81], [82]. Nutritionally stressed animals reduce activity and mobility which in turn compels animals to choose nearby resources that are more accessible, even if these accessible resources are of sub-optimal nutritional quality [4], [12], [83]. Low reproductive output and lower levels of activity increase vulnerability to both ongoing and stochastic threats and suggests a mechanism by which herbivore population dynamics are driven by bottom-up factors. Lunney and colleagues modelled population dynamics in two koala populations in north east NSW and found that small changes in mortality and fertility rates, in particular the death of one breeding female, had a major impact on population viability [71], [84].

Koalas were once common throughout south eastern Australia but a range of factors, particularly hunting for pelts and the clearing of the more fertile lands for agriculture, drastically reduced koala numbers and relegated surviving animals to forests of lower soil fertility in many regions, including in the far south coast region of NSW [41], [44], [85]. Multiple threats continue to impact on koalas including habitat loss, degradation and fragmentation from logging, land clearing, roads and infrastructure [42], [47], [55], [86] as well as environmental impacts from fire and climate change (particularly drought and heatwaves) [46], [87]. Our findings suggest that immediate nutritional concerns are an important priority of south coast koalas making them particularly vulnerable to a range of threats, which in turn implies that nutritional limitations have played a role in constraining koala ecology and populations in the forests of the region. However, as there were no differences in leaf chemistry between trees at occupied and unoccupied plots and because koalas have disappeared from areas where they were once known to persist [42], it is possible that the low-density koala population may spread to parts of the forest not currently occupied if existing threats are controlled.

### Faecal pellets as a proxy of koala visitation

The extremely low density of the Bermagui-Mumbulla koala population (only three koalas seen during the extensive fieldwork) means that koala faecal pellets provide the sole clue of their distribution and habitat use. Faecal pellet surveys have been widely employed to investigate herbivore habitat use, ecology and population distribution in a range of species [88], [89]. Research on browsers in boreal forests of Sweden and in tropical forests of India, have found strong relationships between browse intensity, browse preference and habitat selection and the location and frequency of faecal pellets [90]–[92].

Koala faecal pellets surveys have been employed as a proxy for a range of purposes from surveys of koala abundance and population distribution [93], [94] to developing habitat categorisation for conservation management [46], [50], [56], [95]. Koala faecal pellets have also been widely used to infer habitat and feed tree preferences [56], [96], [97], however their reliability to draw nutritional inferences is still in debate [98]. Koalas are known to visit unpalatable trees, such as *Callitris glaucophylla* in north western NSW, for non-dietary purposes such as thermoregulation [99], [100]. As a result, all studies that record koala tree visitation or use a proxy for feeding such as pellets, rather than observe feeding directly, are likely to include trees that koalas have not fed from. Marsh and colleagues [67] fitted radio and audio-telemetry collars to wild koalas to continuously monitor and quantify feeding activity. They found that koalas ate from 75% of the eucalypt trees they visited and confirmed the nutritional findings of Moore and Foley [62] from the same site whom recorded only koala visitation rather than feeding events. By restricting our study to eucalypts over 15 cm DBH, we ensured that all trees searched for pellets might be considered palatable to koalas. Koalas feed for only 0.9 to 4.7 hours per 24 hours [19], [101], [102], of which 75% occurs at night [67], and deposit pellets continuously over 24 h. However peak deposition times have been found to coincide with peaks in feeding activity [67], [103], thereby increasing the reliability of pellets as indicators of feeding.

A second disadvantage of faecal pellets is that environmental heterogeneity may impact on rates of pellet decay and can lead to false negative results whereby pellets decay and disappear from some trees faster than others [103]. We accounted for this issue by searching our trees twice [98]; first in the initial SAT survey (method described by Phillips and Callaghan [56]), and second during leaf collection. Many trees at Bermagui-Mumbulla had pellets during both these surveys which indicated that koalas had visited them more than once and show a fidelity to these trees. Koalas elsewhere establish stable home ranges and revisit trees that they have marked with their sternal scent gland [101], [104]. Thus, in spite of the disadvantages of using faecal pellets as a proxy for visitation and feeding, a careful statistical design accounts for this potential source of error and allows the identification and differentiation of trees that are important koala habitat resources and to discover vital ecological and nutritional information for the management of cryptic and vulnerable koala populations.

## Conclusions

This study showed that, with a careful experimental design, it is possible to make inferences about populations of herbivores that exist at extremely low densities and thus achieve a better understanding of how nutrition influences herbivore ecology and persistence. We identified a significant tradeoff between nutrients and toxins in the selection of individual trees at a small-spatial scale and found that available N was the key to understanding the selection of some trees. We argue that taxonomic and phenotypic diversity is likely to be important when foraging in habitats of low nutritional quality providing diet choice to tradeoff nutrients and toxins and minimise movement costs. Our findings support the assertion that immediate nutritional concerns are an important priority of folivores in low-quality habitats and imply that nutritional limitations play an important role in constraining folivore populations.
